# Peritumoral lymphatic vessel density and invasion detected with immunohistochemical marker D240 is strongly associated with distant metastasis in breast carcinoma

**DOI:** 10.1186/s12907-017-0041-4

**Published:** 2017-02-10

**Authors:** Nur Fatiha Norhisham, Choi Yen Chong, Sabreena Safuan

**Affiliations:** School of Health Sciences, Universiti Sains Malaysia, 16150 Kubang Kerian, Kelantan, Malaysia

**Keywords:** Lymphatic invasion, D2-40, CD34, Breast carcinoma

## Abstract

**Background:**

Detection of vascular invasion by hematoxylin and eosin staining is the current pathological assessment practice to diagnose breast carcinoma. However, conventional hematoxylin and eosin staining failed to distinguish between blood vessel invasion and lymphatic vessel invasion. Both are important prognostic criteria however with different outcomes. The aim of this study is to distinguish between blood vessel invasion and lymphatic vessel invasion using conventional assessment and immunohistochemical markers. The prognostic significance of both circulatory invasions in invasive breast carcinoma was also investigated.

**Methods:**

Consecutive sections of breast carcinoma samples from 58 patients were stained with CD34 and D240 to stain blood and lymphatic vessels respectively. Hematoxylin and eosin staining was carried out on another consecutive section as conventional staining.

**Results:**

Although blood vessel density is higher in the sections (median = 10.3 vessels) compared to lymphatic vessel density (median = 0.13), vessel invasion is predominantly lymphatic invasion (69.8 and 55.2% respectively). Interestingly, peritumoral lymphatic vessel density and peritumoral lymphatic invasion was significantly associated with distant metastasis (*p* = 0.049 and *p* = 0.05 respectively). The rate of false positive and false negative interpretation by hematoxylin and eosin was 46.7 and 53.3% respectively.

**Conclusions:**

Lymphatic vessel invasion is a strong prognostic markers of breast carcinoma invasion and the use of immunohistochemical markers increase the rate and accuracy of detection.

## Background

Breast carcinoma is one of the leading cancer worldwide with more than 190,000 new cases reported each year in the US [1]. From this figure, the estimated deaths is about 21%. The prognosis of breast cancer is based on the age and menopausal status of the patients, tumor size, histological types and grade, hormone and growth factor receptors and vascular invasion [2].

Currently, vascular invasion is detected microscopically as the presence of tumor cells within the blood or lymphatic vessels using haematoxylin and eosin (H&E) staining [2]. Patients with positive vascular invasion often has poorer prognosis than those with negative invasion. The presence of vascular invasion was also associated with axillary lymph node involvement, systemic relapse and local recurrence [3, 4]. However, conventional assessment of vascular invasion by H&E stained slides was reported to have high rate of false interpretation. Retraction artefacts of the tissue sections tend to be reported as positive invasion while packed tumor cells in a vessel may be missed, leading to false positive and false negative observation of the tissue respectively. There was a low level of concordence (kappa = 0.3) between two observers in reporting vascular invasion of H&E stained tissue section [5]. In addition, H&E staining failed to distinguish between lymphatic vessel invasion and blood vessel invasion in patients’ samples.

D240 is a monoclonal antibody that bind to an O-linked sialoglycoprotein on lymphatic endothelial cells but not on blood endothelial cells. Therefore, D240 antibody has been used as reliable marker to evaluate lymphatic invasion in many research settings [6, 7]. The commercially available D240 antibody binds to a fixation resistant epitope on podoplanin molecules, an integral transmembrane glycoprotein. Ultrastructural analysis revealed the predominat localisation to the luminal surface of lymphatic vessels. CD34 is a common endothelial marker used to detect the presence of blood vessels. This antibody detects a 110 kDa transmembrane glycoprotein expressed on endothelial cells, embryonic fibroblasts and some nervous tissues [8].

The aim of this study is to compare the incidence of lymphatic vessel invasion and blood vessel invasion in breast carcinoma cohort between conventional H&E staining and immunohistochemical staining. The significance of using immunohistochemical markers in relation to adverse clinicopathological criteria is also evaluated.

## Methods

### Patients and specimens

This study was conducted on 58 consecutive formalin fixed paraffin embedded (FFPE) archival specimens of breast carcinoma obtained from Universiti Sains Malaysia Hospital. Clinical characteristics of the patients and tumors are summarized in Table 1.

**Table 1:** Clinicopathological characteristics of breast carcinoma patients and tumors

Clinical criteria	Frequency, n (%)	Clinical criteria	Frequency, n (%)
Age of diagnosis		Recurrence	
≤40	4 (6.9)	No	56 (96.6)
≥40	54 (93.1)	Yes	2 (3.4)
Ethnicity		Estrogen Receptor	
Malay	54 (93.1)	Positive	32 (55.2)
Chinese	4 (6.9)	Negative	26 (44.8)
Tumor Size (mm)		Progesteron Receptor	
<30	11 (19.0)	Positive	28 (48.3)
30 ≤ × < 60	27 (46.6)	Negative	30 (51.7)
>60	17 (29.3)		
No data	3 (5.1)		
Tumor Grading		Lymph nodes involvement	
Grade I	10 (17.2)	1	19 (32.8)
Grade II	27 (46.6)	2-4	6 (10.3)
Grade III	16 (27.6)	>4	16 (27.6)
Not determined	5 (8.6)	Not involved	17 (29.3)
Distant Metastasis		Lymphovascular Invasion (H&E)	
Yes	15 (25.9)	Positive	26 (44.8)
No	37 (63.8)	Negative	28 (48.3)
Not determined	6 (10.3)	Not determined	4 (6.9)
Mastectomy			
Full	10 (17.2)		
Partial	48 (82.8)		

### Immunohistochemistry

Two consecutive FFPE breast carcinoma sections from each patient were stained with CD34 and D240 to assess blood vessels and lymphatic vessels respectively. Staining optimization was conducted on breast sections before using them in the main cohort. 4 μm thick whole sections from each specimen were deparaffinized in two xylene baths for 5 min each and then rehydrated in a series of descending ethanol concentrations (100, 90, 70, 50 and 30% in water for 1 min at each concentration). Antigen retrieval was carried out in 0.01 mol/L-1 sodium citrate buffer (pH6) in a microwave for 20 min; 10 min at full power (750 W) followed by 10 min at low power (450 W). Endogenous hydrogen peroxidase activity was then blocked in 0.3% hydrogen peroxide in methanol for 10 min. Sections were incubated with the primary antibody diluted in antibody diluent (DAKO, Denmark) (1:100 for CD34 and D2-40) for 1 h at room temperature. Unbound primary antibody was washed with TBS prior to the addition of HRP-labeled polymer for 30 min. Sections were then washed and immunohistochemical reactions were developed using 3, 3′ diaminobenzidine (Liquid DAB+ Substrate Chromogen System, DAKO, K3468) for 7 min.

Counterstained with haematoxylin was carried out for 3 min and rinsed off under running tap water. Sections were dehydrated in a series of ascending ethanol concentrations (30,50,70, 90 and 100% in water for 1 min at each concentration), fixed in xylene and mounted with DPX. Sections were left to dry overnight before viewing under the microscope. Tonsil sections were used as both positive and negative controls each time staining was conducted. The procedure as above was applied for positive controls. For negative controls, primary antibody was omitted.

### Microscopic analysis

All microscopic analysis was carried out using light microscope (Olympus, Japan).

### Assessment of microvessel density and lymphatic vessel density

Microvessel density was assessed by counting three hotspots with the highest number of vessels at 100× magnification. The mean value of the hotspots was used in the analysis. For lymphatic vessel density, the positively stained D240 vessels were counted manually across the tissue section. This ensure the accuracy of result as lymphatic vessels are present in much lower density that blood vessel density. For vessel density, both the peritumoral and intratumoral area were counted. Intratumoral was defined as the area within the tumor while peritumoral was defined as the peripheral area at 1 microscopic field of view (×200) from the intratumoral area.

### Assessment of vascular invasion

**Fig. 1:**
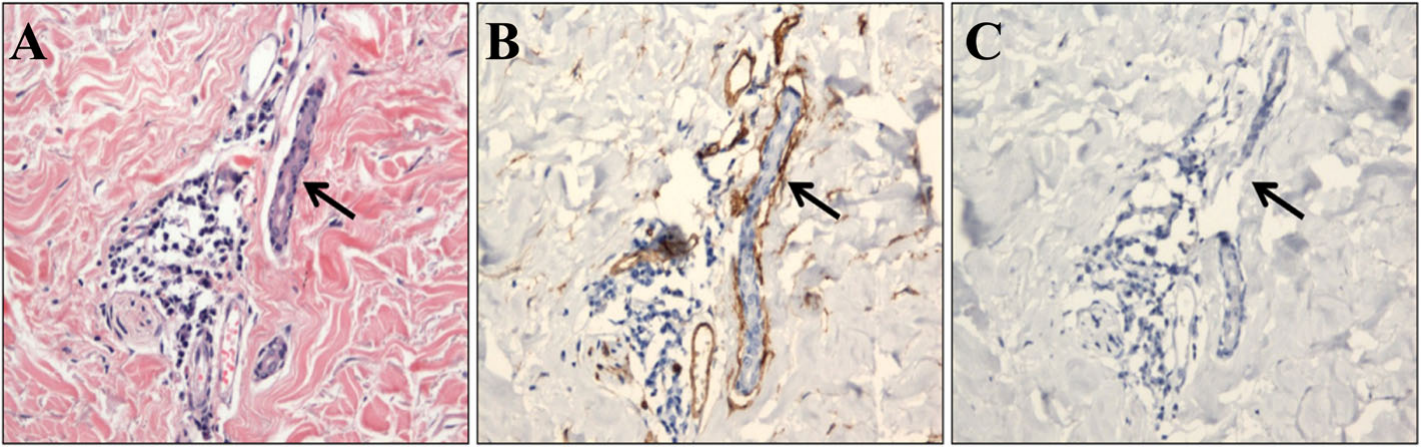
Example of true positive lymphatic vessel invasion determined in D2-40 stained tissue. H&E stained tissue scored as positive lymphovascular invasion (**a**). At the same field of microscopic field view (200× magnification), lymphatic vessel invasion was clearly observed in D2-40 stained tissue (**b**). In CD34 stained tissue (**c**), blood vessel invasion was scored as negative

Figure 1 shows the examples of immunohistochemistry staining with D240 antibody. Lymphatic vessel invasion was identified as the presence of tumor cells within a D2-40 stained vessel. As CD34 can also stain a subset of lymphatic vessels, blood vessel invasion was defined when tumour cells were detected in CD34 positive but D240 negative vessels. The frequency of vascular invasion detected by H&E staining was compared with that detected by IHC. False positive cases were recorded when H&E sections were positive for vascular invasion but negative in IHC. False negatives were recorded when vascular invasion was negative in the H&E sections but positive in IHC. 20% of the specimens were randomly chosen and analysed by second scorer blinded to the results to measure concordance between observers.

### Statistical analysis

Vessels density was classified into high and low category based on the median value. Vessel invasion was divided into negative and positive groups and used as a basis to determine association between all parameters. The relationship between vascular invasion, vessels density and clinical criteria were assessed using chi square test (Fisher exact if the cell count was less than 5). Multivariate analyses were carried out using binomial logistic regression and multiple regression analysis based on the type of variables. Some tumors were not scored because of missing tissue and lack of peritumoral area. P value of less than 0.05 determined significant relationship. Variations between observers were measured using kappa score. Statistical analysis was carried out using IBM SPSS Statistic 24.

## Results

### Distribution of lymphatic and blood vessels

Microvessel density ranged from 5 to 26 vessels with a median of 10.3 vessels. Peritumoral lymphatic vessel density and intratumoral lymphatic vessel density showed a median of 0.052/mm^2^ and 0.078/mm^2^ respectively.

### Frequency of lymphatic and blood vessels invasion

25.7% (*n* = 15) showed no vascular invasion on both intratumoral and peritumoral area. Of the 43 samples with vascular invasion, 69.8% (*n* = 30) showed lymphatic vessel invasion. Of the LVI positive specimens, 33.3% (*n* = 10), 26.7% (*n* = 8) and 40.0% (*n* = 12) were intratumoral invasion, peritumoral invasion and both intratumoral and peritumoral invasion respectively.

**Fig. 2:**
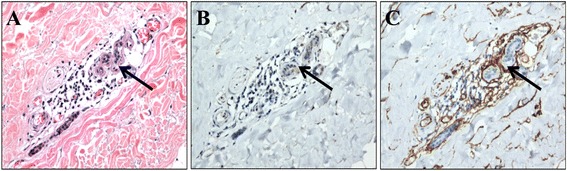
H&E (**a**), D2-40 (**b**) and CD-34 (**c**) staining of consecutive tissue section. This sample was scored as lymphovascular invasion negative by H&E. However, CD34 staining (**c**) clearly showed the presence of tumor cells within CD34 stained vessel and was scored as blood vessel invasion positive. Lymphatic invasion was scored as negative (**b**). Noted that IHC was able to distinguih between lymphatic and blood vessel invasion. (200× magnification)

In comparison to IHC staining, only 55.2% (*n* = 32) showed invasion positive in H&E stained slides. When compared with all cases, 7 cases were false positive while 8 cases showed false negative results. Figure 1 shows the example of positive D2-40 staining which was scored as vascular invasion negative in H&E section. Figure 2 shows the determination of lymphatic vessel invasion positive which was scored as invasion positive in H&E staining. The kappa scores of invasion using immunohistochemical markers between observers were 0.87 and 0.88 for CD34 and D240 respectively. The kappa score of H&E staining between observers was 0.61.

### Association of lymphatic and blood vessel density with clinical criteria

Table 2 shows the association of lymphatic vessel density with clinical criteria. Peritumoral and total lymphatic vessel density was significantly associated with age (*p* = 0.020 and 0.017 respectively) Total lymphatic vessel density was also associated with grade (*p* = 0.018). Interestingly, peritumoral lymphatic vessel density was significantly associated with distant metastasis (*p* = 0.049). Blood vessel density was not associated with all clinical criterias. However, in multivariate analysis none of these variables retain their significant association (peritumoral lymphatic vessel density: age *p* = 0.820, distant metastasis *p* = 0.291 and total lymphatic vessel density: grade *p* = 0.728; age *p* = 0.916).

**Table 2:** Association of lymphatic vessel density with clinical criteria

		Total LVD			Intra-tumoral LVD			Peri-tumoural LVD		
		Low	High	*p*-value	Low	High	*p*-value	Low	High	*p*-value
Age	<40	0 (0.0)	4 (13.3)	**0.020**	2 (7.4)	2 (6.9)	0.941	0 (0.0)	4 (13.8)	**0.017**
	≥40	27 (100.0)	26 (86.7)		25 (92.6)	27 (93.1)		28 (100.0)	2 (86.2)	
Tumor size	<30	2 (7.7)	9 (31.0)	0.080	3 (11.1)	8 (28.6)	0.255	3 (11.1)	8 (28.6)	0.255
	30 ≤ × < 60	15 (57.7)	12 (41.4)		15 (55.6)	12 (42.9)		15 (55.6)	12 (42.9)	
	60 ≥	9 (34.6)	8 (27.6)		9 (33.3)	8 (28.6)		9 (33.3)	8 (28.6)	
Grade	I	2 (7.7)	8 (26.7)	**0.018**	1 (3.7)	9 (31.0)	**0.030**	13 (11.1)	7 (24.1)	0.079
	II	13 (50.0)	14 (46.7)		15 (55.6)	12 (41.4)		14 (51.9)	13 (44.8)	
	III	11 (42.3)	5 (16.7)		10 (37.0)	6 (20.7)		10 (37.0)	6 (20.7)	
	No grade	0	3 (10.0)		1 (3.7)	2 (6.9)		0	3 (10.3)	
Distant metastasis	Yes	7 (25.9)	8 (27.6)	0.989	8 (29.6)	7 (23.3)	0.065	7 (25.0)	8 (27.6)	**0.049**
	No	18 (66.7)	19 (65.5)		19 (70.4)	19 (63.3)		21 (75.0)	17 (58.6)	
	Not determine	2 (7.4)	2 (6.9)		0	4 (13.3)		0	4 (13.8)	
Recurrence	No	26 (96.3)	29 (96.7)	0.940	27 (100)	27 (93.1)	0.1	27 (96.4)	28 (96.6)	0.980
	Yes	1 (3.7)	1 (3.3)		0	2 (6.9)		1 (3.6)	1 (3.4)	
ER	Positive	16 (61.5)	16 (55.2)	0.632	16 (61.5)	15 (53.6)	0.554	17 (63.0)	15 (53.6)	0.480
	Negative	10 (38.5)	13 (44.8)		10 (38.5)	13 (46.4)		10 (37.0)	13 (46.4)	
PR	Positive	15 (55.6)	13 (44.8)	0.422	13 (48.1)	14 (50.0)	0.891	16 (57.1)	12 (42.9)	0.284
	Negative	12 (44.4)	16 (55.2)		14 (51.9)	14 (50.0)		12 (42.9)	16 (57.1)	
Lymph nodes involvement	0	8 (29.6)	11 (37.9)	0.374	8 (30.8)	11 (37.9)	0.678	7 (25.0)	12 (42.9)	0.266
	1	3 (11.1)	3 (10.3)		3 (11.5)	3 (10.3)		4 (14.3)	2 (7.1)	
	2 to 4	6 (22.2)	10 (34.5)		6 (23.1)	10 (31.0)		7 (25)	9 (32.1)	
	>4	10 (37.0)	5 (17.2)		9 (34.6)	6 (20.7)		10 (35.7)	5 (17.9)	

*Bold datas refer to statistically significant association (*p*<0.05)

### Association of lymphatic and blood vessel invasion with clinical criteria

Table 3 shows the association of lymphatic vessel invasion with clinical criteria. Peritumoral lymphatic invasion was significantly associated with age (*p* = 0.012) and distant metastasis (*p* = 0.05). Blood vessel invasion was not significantly associated with all clinical criterias. In multivariate analysis, only age retain the significant association with peritumoral lymphatic vessel invasion (*p* = 0.001).

**Table 3:** Association of lymphatic vessel invasion with clinical criteria

		Intra-tumoral LVI			Peri-tumoral LVI			Total LVI		
		Positive	Negative	*p*-value	Positive	Negative	*p*-value	Positive	Negative	*p*-value
Age	<40	2 (7.1)	2 (7.1)	1.0	4 (14.8)	0 (0)	**0.012**	4 (10.5)	0 (0)	0.065
	≥40	26 (92.9)	26 (92.9)		23 (85.2)	30 (100)		34 (89.5)	19 (100)	
Tumor size	<30	7 (25)	4 (14.8)	0.548	4 (14.8)	7 (25)	0.636	9 (23.7)	2 (11.8)	0.497
	30 ≤ × < 60	12 (42.9)	15 (55.6)		14 (51.9)	13 (46.4)		17 (44.7)	10 (58.8)	
	60 ≥	9 (32.1)	8 (29.6)		9 (33.3)	8 (28.6)		12 (31.6)	5 (29.4)	
Grade	I	3 (10.7)	7 (25.0)	0.387	5 (18.5)	5 (17.2)	0.197	5 (13.2)	5 (27.8)	0.607
	II	14 (50.0)	13 (46.4)		15 (55.6)	12 (41.4)		19 (50)	8 (44.4)	
	III	10 (35.7)	6 (21.4)		7 (25.9)	9 (31.0)		12 (31.6)	4 (22.2)	
	No grade	1 (3.33)	2 (7.1)		0	3 (10.3)		2 (5.3)	1 (5.6)	
Distant metastasis	Yes	9 (32.1)	6 (21.4)	0.432	7 (25.9)	8 (26.7)	**0.05**	11 (28.9)	4 (21.1	0.607
	No	18 (64.3)	19 (67.9)		20 (74.1)	18 (60.0)		25 (65.8)	13 (68.4)	
	Not determine	1 (3.6)	3 (10.7)		0	4 (13.3)		2 (5.3)	2 (10.5)	
Recurrence	No	26 (92.9)	28 (100)	0.92	27 (100)	28 (93.3)	0.105	36 (94.7)	19 (100)	0.198
	Yes	2 (7.1)	0		0	2 (6.7)		2 (5.3)	0	
ER	Positive	16 (59.3)	15 (55.6)	0.632	15 (57.7)	17 (58.6)	0.944	21 (56.8)	11 (61.1)	0.738
	Negative	11 (40.7)	12 (44.4)		11 (42.3)	2 (41.4)		16 (43.2)	7 (38.9)	
PR	Positive	13 (46.4)	14 (51.9)	0.687	13 (48.1)	15 (51.7)	0.789	17 (44.7)	11 (61.1)	0.251
	Negative	15 (53.6)	13 (48.1)		14 (51.9)	14 (48.3)		21 (55.3)	7 (38.9)	
Lymph nodes involvement	0	10 (37.0)	9 (32.1)	0.983	7 (25.9)	12 (41.4)	0.088	13 (35.1)	6 (31.6)	0.763
	1	3 (11.1)	3 (10.7)		1 (3.7)	5 (17.2)		3 (8.1)	3 (15.8)	
	2 to 4	7 (25.9)	8 (28.6)		11 (40.7)	5 (17.2)		10 (27)	6 (31.6)	
	>4	7 (25.9)	8 (28.6)		8 (29.6)	7 (24.1)		11 (29.7)	4 (21.1)	

*Bold datas refer to statistically significant association (*p*<0.05)

## Discussion

The aim of this study was to compare the incidence of lymphatic invasion and blood invasion in breast carcinoma cohort between conventional H&E staining and immunohistochemical staining. It also aimed to investigate the association of lymphatic/blood vessel density and invasion with adverse clinical criteria.

Previous studies showed that lymphatic invasion and blood vessel invasion cannot be distinguished when H&E staining was used in tissue biopsy [8, 9]. The presence of lymphatic and blood endothelial cells specific markers, D2-40 and CD34, respectively made the identification of these vessels possible. It is important to know which type of invasion occurs in individual patients, which has been shown in this study because blood and lymphatic vessel invasion have different prognostic outcomes. In addition, personalized therapy could be designed to cater the needs of every patients based on their invasion routes. Furthermore, this study demonstrated that the concordance score between observers with the usage of immunohistochemical markers is excellent compared to the lower score when reporting vascular invasion of H&E stained tissue section [5]. These showed that immunohistochemical markers could be used in clinical setting with patients samples to minimized the rate of error.

In this study, it was shown that although the frequency of blood vessel density was higher compared to lymphatic vessel density, the incidence of lymphatic vessel invasion exceeded that of blood vessel invasion. Clearly, breast carcinoma preferentially used lymphatic vessels as a route of metastasis. Similar data were also reported by previous study showing that lymphatic vessel invasion incidences exceeded of that blood vessel invasions which were 35 and 16% respectively. Mohammed and colleagues also showed significant invasions of the lymphatics (97%) compared to the blood vessels (3%). These results and our results indicated clearly that breast carcinoma preferentially used lymphatic vessels as a route of metastasis. It was not known what drive tumor cells to invade lymphatic vessels when there are higher number of blood vessel in the tumoral area. One explanation is the anatomical structure of the lymphatic vessel itself which would not offer a significant barrier for the entry of tumor cells [10]. The lack of basement membrane and supporting structure in the lymphatic vasculature may ease the intravasation of tumor cells into the lymphatic circulation. In contrary, lymphatic endothelium might also play an active role in tumor cells recruitment. They may secrete chemokines that attracted the tumor cells to the lymphatic capillaries. Chemokines receptor-ligand relationships are important to regulate leukocytes trafficking and this relationship was hypothesized to be exploited by cancer cells to modulate entry into the lymphatic circulation [11, 12].

The presence of intratumoral lymphatic vessel invasion has been debatable as it was thought that lymphatic vessel could not penetrate the high pressure environment inside the tumor mass. With the discovery of new molecular markers and better imaging systems, the presence of intratumoral lymphatics has been reported [13]. In this study, we reported the presence of intratumoral lymphatic vessel invasion however no significant association was observed with any clinical criteria under study. Previous study has demonstrated the association between intratumoral lymphatic invasion with markers of aggressiveness [14] which was not observed in this cohort perhaps due to the number of samples used.

The clear association observed between peritumoral lymphatic vessel density and invasion with distant metastasis indicates that peritumoral lymphatic vessels have important role in breast carcinoma metastasis to secondary organs. Distant metastasis is a major factor that lead to poor prognosis of cancer patients. Statistics shows that 90% of cancer-related death occurs as a result of metastasis [15]. The highly significant association between these two variables may indicate that targeted therapy directed to peritumoral density could be designed to reduce metastatic dissemination of cancer cells especially in breast carcinoma patients.

## Conclusions

In conclusion, although blood vessel density in breast carcinoma patients is higher compared to lymphatic vessel density, vascular invasion in breast carcinoma is predominantly lymphatic vessel invasion. The fact that peritumoral lymphatic vessel invasion was strongly correlated with distant metastasis shows that it is a strong predictor of breast carcinoma outcome. This study should be repeated in larger cohort with relapse-free survival and overall survival data. We strongly reccommended that IHC markers be used alongside H&E staining to improve the detection rate and false interpretation of tissue samples.

## Abbreviations

BVI: Blood vessel invasion
FFPE: Formalin fixed paraffin embedded
H&E: Hematoxylin and eosin
LVI: Lymphatic vessel invasion
